# The First Bite— Profiling the Predatosome in the Bacterial Pathogen *Bdellovibrio*

**DOI:** 10.1371/journal.pone.0008599

**Published:** 2010-01-06

**Authors:** Carey Lambert, Chien-Yi Chang, Michael J. Capeness, R. Elizabeth Sockett

**Affiliations:** Institute of Genetics, School of Biology, Nottingham University, Queen's Medical Centre, Nottingham, United Kingdom; Baylor College of Medicine, United States of America

## Abstract

*Bdellovibrio bacteriovorus* is a Gram-negative bacterium that is a pathogen of other Gram-negative bacteria, including many bacteria which are pathogens of humans, animals and plants. As such *Bdellovibrio* has potential as a biocontrol agent, or living antibiotic. *B. bacteriovorus* HD100 has a large genome and it is not yet known which of it encodes the molecular machinery and genetic control of predatory processes. We have tried to fill this knowledge-gap using mixtures of predator and prey mRNAs to monitor changes in *Bdellovibrio* gene expression at a timepoint of early-stage prey infection and prey killing in comparison to control cultures of predator and prey alone and also in comparison to *Bdellovibrio* growing axenically (in a prey-or host independent “HI” manner) on artificial media containing peptone and tryptone. From this we have highlighted genes of the early predatosome with predicted roles in prey killing and digestion and have gained insights into possible regulatory mechanisms as *Bdellovibrio* enter and establish within the prey bdelloplast. Approximately seven percent of all *Bdellovibrio* genes were significantly up-regulated at 30 minutes of infection- but not in HI growth- implicating the role of these genes in prey digestion. Five percent were down-regulated significantly, implicating their role in free-swimming, attack-phase physiology. This study gives the first post- genomic insight into the predatory process and reveals some of the important genes that *Bdellovibrio* expresses inside the prey bacterium during the initial attack.

## Introduction

**Figure 1:**
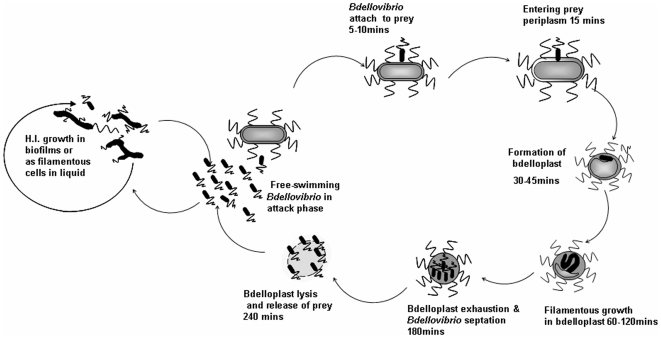
Predatory and HI growth cycles of *Bdellovibrio*. Attack-phase cells invading *E. coli* prey undergo a predatory growth cycle and replicate, being released some 4 hours later. Timing of the predatory cycle events are shown, including the 30 minute predation stage when *Bdellovibrio* are in the bdelloplast. Axenic growth of pleiomorphic sessile HI cells occurs on protein-rich media for cells derived from attack-phase populations.

*Bdellovibrio bacteriovorus* are predatory delta Proteobacteria which invade the periplasm of other Gram-negative bacteria and attach to their inner membrane forming an infective structure called a bdelloplast. In this structure the prey peptidoglycan is modified so the bdelloplast does not burst, but accommodates the growing predator; the cellular constituents of the prey bacterium are degraded to monomers which are taken up and used to fuel growth and division of the *Bdellovibrio* (Figure 1). The gene products required for the initial invasive predatory processes have not been extensively studied but the genome sequencing of *B. bacteriovorus* HD100 [1] revealed a genome of 3.85Mb, including a core genome similar to that of non-predatory bacteria and some 40% of the genome comprising a potential predicted “predatosome” of genes, encoding both hydrolytic products that may be employed in prey degradation, and genes that may be required specifically for host predation and thus are not conserved across the Proteobacteria.

**Figure 2:**
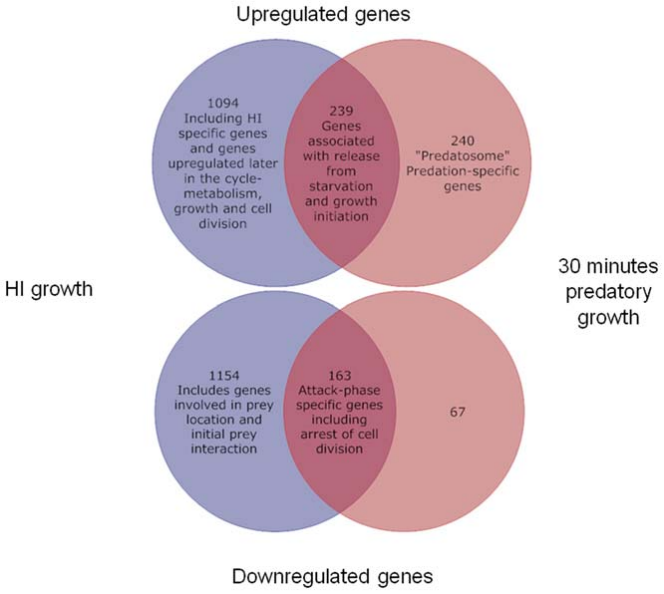
Combining Attack-phase, HI and Predatory Datasets. Venn diagram produced by combining our datasets in a comparison of genes up or down-regulated in 30 minutes predation versus attack-phase and those up or down regulated in HI growth versus attack phase. This allows identification of gene expression patterns specific to each growth state- 30 minutes predation, attack-phase outside prey, or axenic HI growth without prey. For example 240 genes that are specifically up-regulated only upon 30 minutes predation, compared to attack phase; are delineated away from a set, of 239 “release from starvation and growth initiation” genes that are up-regulated both in predation and HI growth, compared to non-replicative attack-phase *Bdellovibrio*.

By combining careful semi-synchronous culturing of prey and predators with RNA preparations we have been able to study the transcriptome of *Bdellovibrio* at 30 minutes of predatory interaction, a key point in predation, as the *Bdellovibrio* enters the prey periplasm and establishes itself by attaching to the prey cytoplasmic membrane and killing the prey. Other timepoints in the predatory process will also be fascinating to study but would be difficult to synchronise, so here we report genes whose products are involved in the early “predatory bite” of *Bdellovibrio* at 30 minutes of interaction with prey cells. We also have identified those genes that are involved in the motile, “hungry”, non-replicative, attack-phase life-style of *Bdellovibrio*, which it adopts when seeking out prey, and that are down regulated when the bacteria enter prey and establish a bdelloplast. As *Bdellovibrio* begin replication after they enter prey we also profiled gene expression in *Bdellovibrio* that were growing axenically on lab media, in the so-called prey/host-independent “HI” growth mode, again in comparison to the attack-phase condition used above (Figure 1). We then combined and compared the data sets with each other to delineate *Bdellovibrio* genes whose expression, at 30 minutes predation, was dedicated to predatory processes rather than just growth *per se* (Figure 2). We also were able to define genes that were expressed specifically in HI growth and not in either predatory attack phase or 30 minutes of prey-invasion.

Our data illuminate, for the first time across the genome, some of the molecular inventory required for the predatory lifestyle of *Bdellovibrio*. Some of these differentially expressed genes have been previously found by ourselves and others in directed studies of particular genes, or random mutagenesis, to be important in predation or HI growth and many others are newly associated with discrete phases in the predatory process.

## Materials and Methods

### Culture Conditions for Predatory Infections

*Escherichia coli* S17-1 prey and *Bdellovibrio bacteriovorus* HD100 were grown as described previously [2]. To establish near synchronicity of infection, predatory co-cultures were sub-cultured in 50ml Ca/HEPES buffer for 3 days at 24 hour intervals before the resultant *Bdellovibrio* were used to inoculate a 1L culture of Ca/HEPES buffer with 60ml of an overnight (18 hour) culture of *E. coli* S17-1 prey. The resulting attack phase *Bdellovibrio* after 24h incubation were concentrated 10 times by centrifugation and incubated alongside 100ml of overnight cultured *E. coli* adjusted to OD_600_ of 1.0 in Ca/HEPES buffer for 3 hours with shaking at 200rpm at 29°C to stabilise gene expression. Colony counts of *E. coli* and plaque counts of *Bdellovibrio* showed that there was no significant loss of viability during incubation in these stabilisation conditions. 50ml of this *Bdellovibrio* attack-phase culture were mixed with 40ml of the *E. coli* culture and 30ml Ca/HEPES buffer for the infection. Control infections of *Bdellovibrio* only and *E. coli* only were carried out concurrently. At 30 minutes post-infection or control, 10ml samples were taken into 1% phenol 19% ethanol (final v/v), incubated at 4°C for 45 minutes, spun down at 3320g for 10 minutes at 4°C before the pellet being stored at −80°C for later RNA purification [2].

### Choice and Verification of HI Strain and Culture Conditions for HI

*Bdellovibrio* HI strains are known to vary considerably in cell morphology, motility, pigmentation, growth rate and predation ability so it was important to choose wisely in our expression profiling. For the HI array experiments we carefully chose an HI strain HID13 which we isolated (alongside other HI strains) and grew axenically from the predatory HD100 type strain by differential filtration and growth on PY media [3]. HI strains of *Bdellovibrio* are notorious for showing a diversity of cell length, pigmentation, motility and predation capabilities and many of them have a mutation in a gene Bd0108 at the so-called *hit* locus, a locus associated with axenic growth. HID13 was chosen, from a range of other HI strains, as it showed flagellar motility, a median typical cell length for an HI culture, proportion of elongated cells, and retained predatory ability as determined by the YPSC overlay technique. We determined that it had a single point mutation at the start codon of Bd0108 causing ATG→ATA. After we determined the expression profile for HID13 and verified expression levels by QPCR for representative (up-, down, and unchanged) genes we also checked the expression of those and other genes in matched quantities of mRNA from other HI strains we had isolated to verify that the effects were not HID13-specific. The other HI strains we used were HID22 which has a 42bp deletion in Bd0108 and much longer cells on average; HID2 which have a wild-type Bd0108 sequence but a higher proportion of small motile cells than most other HI strains and HID26 which have a wild-type Bd0108 sequence but have a larger proportion of sphearoplasting-morphology cells than typical. HI strains are also reported to change phenotype upon prolonged HI culturing [4], so copious frozen stocks of each strain were prepared as soon as possible after isolating and initial growth and all experiments were carried out on strains grown from these frozen stocks with the minimum length of culturing time.

Host independent (HI) strain HID13 of *Bdellovibrio bacteriovorus* were streaked onto PY plates from frozen stocks and incubated for 3–5 days. A sample from these plates was used as an inoculum for a 2ml PY broth culture which was incubated overnight with 200rpm shaking at 29°C and then increased to 50ml before further overnight incubation. This culture was then back-diluted to OD_600_ of 0.1 and then grown to log phase (OD_600_ of 0.6±0.1). 4ml samples were taken into 1% phenol 19% ethanol (final v/v), incubated at 4°C for 45 minutes, spun down at 3320g for 10 minutes at 4°C before the pellet being stored at −80°C [2].

### RNA Preparations

RNA was prepared from the frozen bacterial pellets using modifications to the Promega SV total RNA isolation kit protocol, published elsewhere [2] except that double the initial volumes were used to take into account the larger bacterial pellets i.e. 200µl starting volume. Several RNA preparations from the same infection were pooled for array analysis.

### Oligonucleotide Arrays and Data Analysis

For the HI array experiments, matched amounts (10µg) of RNA were used from attack phase *Bdellovibrio bacteriovorus* HD100 and Host-Independently grown *B. bacteriovorus* HID13. For the predatory array experiments the presence of the *E. coli* RNA in the infection resulted in this sample having 5.12 times the amount of total RNA compared to the *Bdellovibrio bacteriovorus* HD100 only control and so, in order that equivalent amounts of *Bdellovibrio* RNA was labelled for array analysis, 10µg of *Bdellovibrio* only RNA and 51.2µg infection RNA were used for labelling for each array hybridisation. Oligonucleotide arrays were produced by NimbleGen Systems Inc. and their expression labelling and hybridisation service was used. Three independent experiments were carried out and the log ratio (base 2) of the normalised data provided by NimbleGen was used for statistical analysis using *t*-test in the package MEV [5]. A paired *t*-test with a threshold *p*-value of 0.05 was used. The normalised data were also analysed using SAM with a cut-off of 1.3-fold change in expression and a delta of 0.0 which resulted in a median false discovery rate of 0.11%. For stringency and confidence in the data, only those genes called significant in both of these analyses were considered. The predatory data discussed in this publication have been deposited in NCBIs Gene Expression Omnibus (GEO, http://www.ncbi.nlm.nih.gov/geo/) and are accessible through GEO Series accession number GSE9269. The HI data discussed in this publication are accessible through GEO Series accession number GPL8539. Datasets were compared from all three conditions (attack-phase : predatory 30 minutes : HI) to derive the Venn diagram shown in Figure 2.

For the 30 minutes predation data, the nucleotide sequence of each significantly differentially expressed gene was compared to the Colibase [6] sequence database, by BLASTn analysis, to determine if there was any significant sequence identity with the *E. coli* genes. This was important as this data set, unlike the HI and attack-phase datasets, was produced against a background of *E. coli* prey RNA. Only 19 genes showed significant (E<0.001) identity enough to have sufficiently few mismatches to potentially bind the 25-mer probes of the arrays in the stringent hybridization conditions used. Of the genes discussed in this work, only the ATP synthase genes, RNA polymerase genes and Bd0099 are included in these and so it should be borne in mind that these may be false positives. RT-PCR on the ATP synthase genes however- with controls of *E. coli* only RNA giving a negative result- showed that this is unlikely.

### QPCR Analysis to Verify Gene Expression Levels

Quantitative real-time RT-PCR was carried out on Stratagene MX3005P or MX4000 machines, using the Statagene Full Velocity SYBR Green QRT-PCR kit in one-step reactions as described previously [7]. RNA samples were prepared as detailed above and serial dilutions of these were used for QPCR with primers designed to anneal to transcripts from two genes that were up-regulated upon 30 minutes predation (Bd1904, Bd0416), two that were down-regulated (Bd2620, Bd0659) and two that were not significantly changed for the 30 minutes predation condition (*sdhB* :Bd0026, *pilA*: Bd1290). We also used primers designed to anneal to transcripts from two genes that were up-regulated (*narL* :Bd2837, Bd1476), two down-regulated (Bd2462, Bd0367) and two not significantly changed for the HI growth condition (*dnaK*: Bd1298, Bd1168). Absolute expression was calculated against a standard curve of a template of extracted PCR product and the ratios of expression in the two samples were compared. At least two independent experiments were carried out and included relevant controls such as no template, no reverse transcriptase and also with *E. coli* RNA alone as a template. A *t*-test was carried out on each set of data to ascertain significance. The PCR products were sequenced to confirm the specificity of the reactions.

Semi quantitative end-point RT-PCR, used to compare expression in different HI strains to that of HID13, was as described elsewhere [2], except with matched amounts of RNA (to 10ng per µl as determined by nanodrop spectrophotometer) from several HI strains and host dependent strains from attack phase or 4 hours post infection (both stages contain negligible amounts of *E. coli* prey RNA). Suitable controls were carried out with no template, no RT and controls with genomic DNA or *E. coli* RNA as template.

## Results and Discussion

### Microarray Analysis and Dataset Comparison

**Figure 3:**
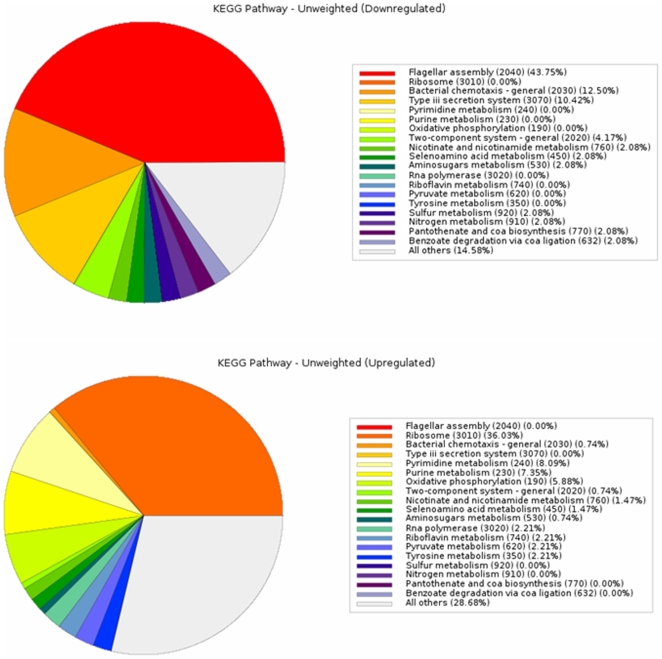
Up- and Down-regulated genes from attack-phase to predatory come from very different KEGG categories. Pie charts showing the very different category distributions, of significantly down regulated or up-regulated *Bdellovibrio* genes upon the shift from attack phase to 30 minutes predation. (Only genes that have KEGG categories are shown, organised by those categories). Type iii secretion system category refers only to flagellar genes as *Bdellovibrio* has no other Type III system).

We considered genes that were significantly differentially regulated by both statistical criteria mentioned in the methods. In the attack-phase to 30 minutes predation shift, this gave 479 genes (13% of the genome) that were up-regulated and 230 genes (6.4%) that were down-regulated by 1.3 fold or higher upon 30 minutes of *Bdellovibrio*-prey interaction ( a growth condition hereafter referred to as “predatory”) compared to attack-phase *Bdellovibrio*. Although many of the differentially expressed genes were hypotheticals, those with KEGG categories fell into markedly different categories for up- versus down-regulated (Figure 3). Many genes were highly up-regulated with 62 of the 479 being up-regulated between 5- and 16-fold in predatory versus attack-phase. Some of these genes lie within clusters that also seem to be up-regulated but fall just below the 1.3 factor cut-off and statistical analyses we apply here, and so their surrounding genes were taken into consideration during data interpretation. It must be remembered that in our predatory samples large amounts of prey RNA were present and beginning to be degraded by the *Bdellovibrio*. Although we controlled against cross reactivity for our array data, as mentioned above, the background of RNA may have acted as a non-specific blocking agent or buffer reducing signal. This is why we have taken an expression cut-off of genes that are 1.3 fold up-regulated and above, although many of the genes we studied were very highly differentially regulated. Hughes and co-workers reporting in Cell [8] verified the validity and significance of genes expressed at cut-off levels of 1.2 and above in expression studies that they carried out on *E. coli*.

For the HI growth versus attack-phase comparison the same statistical approach and cut-off value, resulted in 1333 genes (37.2%) up-regulated and 1317 genes (36.7%) down-regulated from attack phase to HI growth. Again there were high levels of up-regulation with 212 genes being between 10- and 76-fold up-regulated in HI compared to attack phase. While this appears to be a very large proportion of the genome differentially regulated, this is not unexpected as the HI growth phase includes growth, development, genome replication and cell division whilst the attack phase is a much more specialised, non-replicative phase of motility and prey location and so the genes required for these stages are likely to be very different. As such, it is difficult to determine which genes are important for each individual function. Comparisons between attack phase and HI cultures are not prone to the RNA dilution/cross reaction effects discussed for the predatory situation above and mentioned by others [9] so differential expression between these two states could be further tested and refined. Future studies could test the HI synchronisation techniques reported by Jurkevitch and co-workers to determine whether the HI-specific dataset is altered at particular timepoints in HI growth [10].

However, by comparing the attack phase-HI dataset to that above, where gene expression changes caused by change from attack phase to 30 minutes of predatory intraperiplasmic growth by *Bdellovibrio* were measured; many interesting subsets of differentially regulated genes can be identified and putative activities assigned.

Figure 2 shows the overlap and differences between datasets comparing attack phase to HI and attack phase to predatory growth in prey periplasm. This shows 163 genes (4.5%) down-regulated in both datasets and 67 genes (1.9%) down-regulated in the transition to predatory growth alone; together these 230 represent genes specific to the non-replicative, highly motile, mono-flagellate attack phase cell type. The 239 genes (6.6%) up-regulated in both predatory and HI datasets likely represents genes involved in release from the attack phase “starvation” conditions and growth and development initiation. Of particular interest are the 240 genes (6.6%) which are specifically up-regulated upon predation and NOT up-regulated during HI growth suggesting that they are part of a “predatosome” of genes specifically involved in the interaction with prey cells. These data further support the idea that these genes are important in predation, as was postulated by their up-regulation upon entry to the bdelloplast. Many genes (1094) (30%) are up-regulated in HI, but not upon predatory growth in prey bdelloplast. Whilst most of these are likely to be involved in metabolism, growth, genome replication and cell division that is not yet taking place upon 30 minutes post infection, some of these are likely HI-specific genes and further analysis by RT-PCR on some of them has confirmed this. The genes belonging to each group are listed in Figure S1.

In order to ascertain that some genes were up-regulated in HI growth specifically, and not just later in the predatory cycle, expression of *narL*;Bd2837, Bd1640 and Bd0646 was monitored throughout the predatory cycle and RNA samples from the point of highest expression were used in matched amounts to compare to HID13 RNA. In all cases, the samples were 3 hour or 4 hour post-infection and so had negligible amounts of prey RNA remaining at this point of the predatory cycle [11], and in all cases, there appeared to be higher expression in the HI growth conditions showing that these genes are indeed expressed more highly in these conditions (Data not shown).

**Figure 4:**
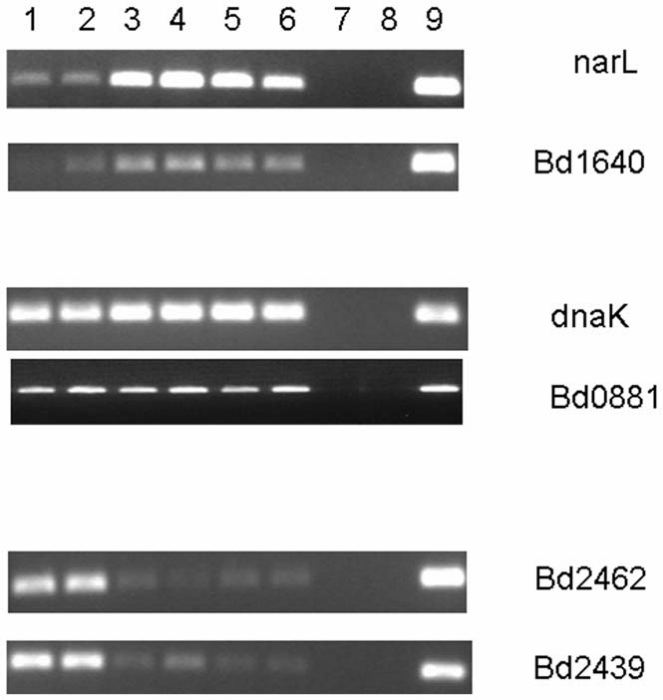
RT-PCR to show that differential expression is not HI-strain specific. RT-PCR on matched amounts of RNA to compare host-dependent attack phase (sample 1) and 4 hours post-infection (2) with 4 different HI strains HID13 (3), HID2 (4), HID22 (5) and HID26 (6) along with controls of *E. coli* prey RNA (7), no template (8) and HD100 genomic DNA as template (9). Primers were designed against 2 genes upregulated in HI phase (*narL* and Bd1640), 2 genes constitutively expressed in both phases (*dnaK* and Bd0881) and 2 genes with lower expression in the HI phase (Bd2462 and Bd2439). The fact that all 4 strains show the same expression patterns confirms that the results obtained were a consequence of the HI phenotype in general and not specific to the strain HID13 used for the arrays.

As mentioned in materials and methods, we verified that our transcriptional results were not specific to the chosen HI strain by carrying out RT-PCR for genes called as differentially regulated or called as stable in RNA from 3 other HI strains (Figure 4).

### Confirmation of Array Results by QPCR

**Figure 5:**
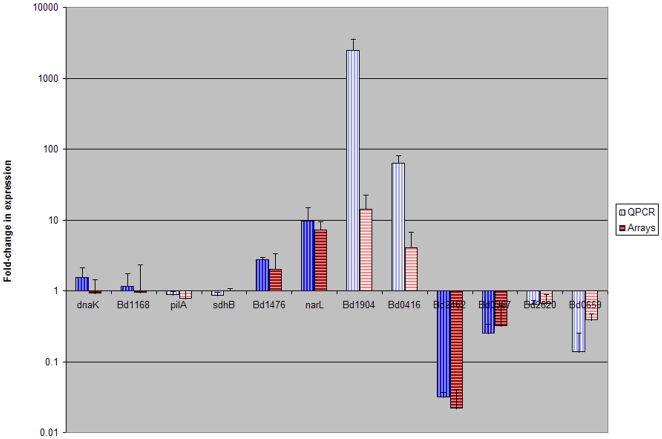
QRT-PCR results confirming the array results displayed on a log scale. T-test on the QRT-PCR data also showed that *sdhB* and *pilA* were not significantly differentially regulated in the transition from attack phase to predatory conditions and that *dnaK* and Bd1168 were not significantly differentially regulated in the transition from attack phase to HI conditions. It shows that Bd1904, Bd0416, Bd0659, and Bd2620 were significantly differentially regulated in the transition from attack phase to predatory and that *narL*, Bd1476, Bd2462 and Bd0367 were significantly differentially regulated in the transition from attack phase to HI. (T-test results confirmed the differences in expression between differentially regulated genes to be significant and that there were no significant differences in expression levels for those genes called as not differentially expressed in the array data). Darker colours are for genes confirming the HI array data, lighter colours for the 30 minutes predation experiment.

The array results were confirmed for the predatory and HI data sets by performing QPCR on two genes that were up-regulated (Bd1904, Bd0416 for predatory and *narL*:Bd2837, Bd1476 for HI; Figure 5), two down-regulated (Bd2620, Bd0659 for predatory and Bd2462, Bd0367 for HI; Figure 5) and two not significantly changed in the HI or predatory dataset (*sdhB*:Bd0026, *pilA*: Bd1290 for predatory dataset *and dnaK*: Bd1298, Bd1168 for HI dataset; Figure 5). T-tests on the QPCR data showed that the latter two pairs of genes were, as expected, not significantly differentially regulated in the appropriate HI or predatory condition, whilst the others were, demonstrating that the two independent methods were in agreement of statistical significance.

The differentially regulated genes at predatory conditions and HI conditions and their functional categories are discussed below.

### Genes Up-Regulated in Both HI and Predatory Datasets

Predominantly, the 239 genes that are significantly up-regulated in going from the non-replicative attack-phase to either HI or predatory conditions are, as would be expected, RNA polymerase genes including Bd2950, Bd2984 and *rpoD* sigma factor Bd0242; transcription factors and ribosomal genes and translation factors including anti-termination factors such as *nusG* Bd2992 [12] and *greA* [13], presumably to deal with the extra transcription. Similarly, *nusA* Bd1546 is an essential transcription elongation factor [14]. Genes encoding translation elongation proteins Tuf Bd2994 [15] and Ts Bd3782 [16] are up-regulated presumably to deal with the extra translational load as is Bd0069 the peptide chain release factor I [17] which mediates translation termination. These functions will be associated with producing hydrolytic enzymes to degrade prey components in the bdelloplast, or complex macromolecules in HI growth media, and also simply to begin to grow cellular mass and divide in HI cells (at 30 minutes predation growth will be beginning but division not yet proceeding). In addition ATP synthase genes are up-regulated to support such anabolism, secreted-enzyme synthesis and export and solute uptake via, in some cases ATP-dependent ABC transporters such as Bd0995, Bd1221 and Bd3637.

GroES- EL chaperone and Bd3764 trigger factor [18] genes are up-regulated to assist with the waves of protein synthesis that occur and a few genes associated with cell wall/membrane/electron transport chain biogenesis are induced including Bd0468, Bd1940 and Bd3487. As this “up in both” dataset has, by definition, to include genes up-regulated at 30 minutes predation (and HI) then not enough predatory growth will have occurred by 30 minutes to require chromosomal replication and cell division, so it is not surprising that only one DNA polymerase subunit gene Bd3834 is called as up-regulated, other DNA replication encoding genes are up-regulated in the HI-specific gene set. It is interesting that Bd0039 encoding a site-specific invertase is induced in both HI and predatory datasets, possibly indicating the role of invertible promoters in the exit from attack-phase [19].

Transporters, both exporters of effector proteins and enzymes and importers of growth substrates are important to the predatory process as well as the extracellular degradation of complex protein molecules and monomer uptake for HI growth [20]. The prey cytoplasmic components remain compartmentalised from the *Bdellovibrio* in the bdelloplast so transport is required for degradation too. We found that Bd2955 encoding the SecY preprotein translocase [21] is significantly up-regulated in both datasets and other *sec* genes are up-regulated but just miss our statistical significance test, reiterating the importance of Sec system in secreting the many degradative enzymes into the prey cytoplasm. Twin arginine transport gene Bd2196 which encodes a homologue of the TatA pore protein, named TatE [22] is also up-regulated suggesting that some predatory, as well as HI, proteins may be secreted by this alternative transport system. This supports an unpublished observation from our laboratory (C-Y Chang in preparation) that a *tatE* deletion strain had both predatory and HI growth defects. Other up-regulated genes include *comL* [23] encoding a “competence protein” involved in DNA uptake, this is an interesting up-regulated gene for a bacterium that takes up prey DNA at least in a degraded form; does this protein allow uptake of any prey DNA segments, after endonuclease activity, into predator; does it allow transformation of *Bdellovibrio* in the HI, but not attack-phase state? Only 4 other predicted membrane transport genes of a predicted 244 in the genome were up-regulated in both HI and predatory datasets, Bd2512 for glycerol-3-phosphate transport, Bd1221 for dipeptide transport and two other ABC transporters. It seems likely that many more will be up-regulated in the predatory state soon after the 30 minute point as breakdown products from the prey are detected. Indeed this has been shown to be the case, later in the predation process, for the maltoporin *lamB* [24].

### Genes Up-Regulated in Predatory, but Not HI, Datasets

This category of 240 genes are very interesting as they potentially exclude those genes simply involved with release from attack-phase into growth, namely they should be part of the “predatosome” of predatorily specific genes. This group of genes is dominated by 174 genes encoding hypothetical proteins, presumably cryptic members of the predatosome and very worthy of our mutagenesis and tagging, as a *Bdellovibrio* community, to test their predatory roles.

After the highly upregulated hypothetical genes, a large operon of genes Bd0412–Bd0420 is highly predatorily up-regulated (4–7 fold) at 30 minutes predation, but not under HI growth and its genes Bd0417–0420, which are clustered after hypotheticals Bd0412–0416, are homologous to those encoding a TonB system and are annotated as encoding adventurous gliding motility (Agl) proteins due to homology to those in the Myxobacteria [25]. In the Myxobacteria, which are delta proteobacterial “cousins” of *Bdellovibrio*, the *agl* gene products may be involved in a contact sensitive process energised by the TonB-like component (TonB systems energise outer membrane transport processes in other bacteria). Thus it is possible that Bd0412–0420 are genes with products involved in prey contact and establishment of *Bdellovibrio* in the periplasm or the cross-membrane-energised secretion or uptake of molecules during predation. Interestingly, as is discussed later in genes up-regulated in HI but not predatorily, there are 3 other homologous *tonB*-like/*agl* operons in the *Bdellovibrio* genome (Bd0828–Bd0838, Bd1473–Bd1483 and Bd2368–Bd2377) with different regulatory patterns.

Some of the many sensor-regulator genes of *Bdellovibrio* are specifically predatorily up-regulated to presumably sense developing conditions in the bdelloplast and programme later regulatory events such as the secretion of new waves of hydrolytic enzymes, *Bdellovibrio* septation and prey cell lysis in response to the available prey nutrient levels. These include transcriptional regulator genes Bd0136, Bd1634, Bd3063, two-component sensor-kinase genes Bd3359, Bd3360 and also Bd2320 a CarD like transcriptional regulator [26].

Several peptidoglycan metabolising genes are up-regulated in predatory conditions and not HI. These may be employed for remodelling *Bdellovibrio* to squeeze through the outer-membrane pore in the prey cell, for remodelling the growing *Bdellovibrio* sacculus as the cell begins to replicate, or for metabolising prey peptidoglycan (including de-cross-linking it), to provide a stable but spacious bdelloplast structure or to liberate cell wall monomers for *Bdellovibrio* growth; all of these activities will be occurring, especially the former and the latter, as the *Bdellovibrio* settles into the prey periplasm. Bd0816 and Bd3459 genes are up-regulated 5.9 and 5.4 fold upon predation. These encode the D-ala-D-ala carboxypeptidases which cleave the peptide crosslinks in peptidoglycan between D amino-acids, an activity that is recorded in old physiological papers when *Bdellovibrio* round up prey cells and form the bdelloplast making space in the periplasm to settle into [27]. Also highly up-regulated were genes Bd3575 encoding a soluble lytic murein transglycosylase which could degrade peptidyglycan backbone strands, Bd1358 encoding a putative peptidoglycan binding protein, and Bd3279 encoding a polysaccharide de-acetylase. De-acetylation of peptidoglycan is another activity already biochemically verified when *Bdellovibrio* act upon prey cell walls in the bdelloplasting process [28]. The roles of these peptidoglycan remodelling genes in predation is currently under further study in our lab.

As mentioned earlier, predatory invasion brings with it a sudden burst in protein synthesis and chaperone gene up-regulation. In a related role peptidyl prolyl isomerase Bd1903 which encodes a protein which contributes to the folding of proline-containing proteins [29] is predatorily up-regulated while peptidyl prolyl isomerase Bd0722 expression falls in the shift from attack phase to predatory and to HI growth. The peptidyl prolyl isomerases are periplasmic proteins of Gram-negative bacteria. That one falls as the other rises may indicate altered specificities for different predatory-versus-free-living proteins and altered accessory proteins involved in binding to them to carry out the cis-trans isomerisation and conformational alterations.

In addition to the ATP synthase up-regulated in both predatory and HI conditions, genes encoding other components of the electron transport chain were highly predatorily up-regulated including Bd1938 encoding cysteine desulphurase and Bd0187 and Bd0188 encoding transport proteins, all involved in Fe-S cluster assembly [30].

### Genes Encoding Enzymes for Potential Prey Degradation

As this early timepoint in predation only a few genes of the very large arsenal encoding hydrolytic enzymes are de-repressed as, although the *Bdellovibrio* have entered the prey periplasm, degradation of the prey cell contents is just beginning. These early-expressed gene products are predominantly for protein degradation but those expressed at this early time point of infection represent a very small fraction of those in the genome. Only 11 of the 150 putative proteases encoded in the *Bdellovibrio* genome are up-regulated at this stage, representing several different classes including genes predicted to encode peptidases, a Zn-metallo-protease, a cysteine-protease and several serine protease homologues. Genes predicted to encode two endonucleases (Bd1934 and Bd1244) of 20 putative nucleases and a helicase (Bd1370), and two other hydrolases of different classes of a putative total of 89, are up-regulated in the predatory gene-set. The products of these early expressed protease genes may well be contributing to penetration of prey membranes rather than extensive cytoplasmic hydrolysis, although the endonucleases are predicted to be exported so are likely to be involved in early breakdown of the prey DNA, before the later expression and action of exonucleases. Some genes that might protect *Bdellovibrio* from free radicals generated in the dying prey cell bdelloplast are induced in the shift to predatory growth, for example Bd0355 encodes a thioredoxin like protein, Bd1626 encodes an isopentenyl pyrophosphate isomerase, required to synthesise the starting materials for carotenoids and long chain isoprenoids [31]. These will afford protection from oxidative damage by free radicals in the periplasm of prey.

### Up-Regulated Genes Unique to Predatory Bacteria

**Figure 6:**
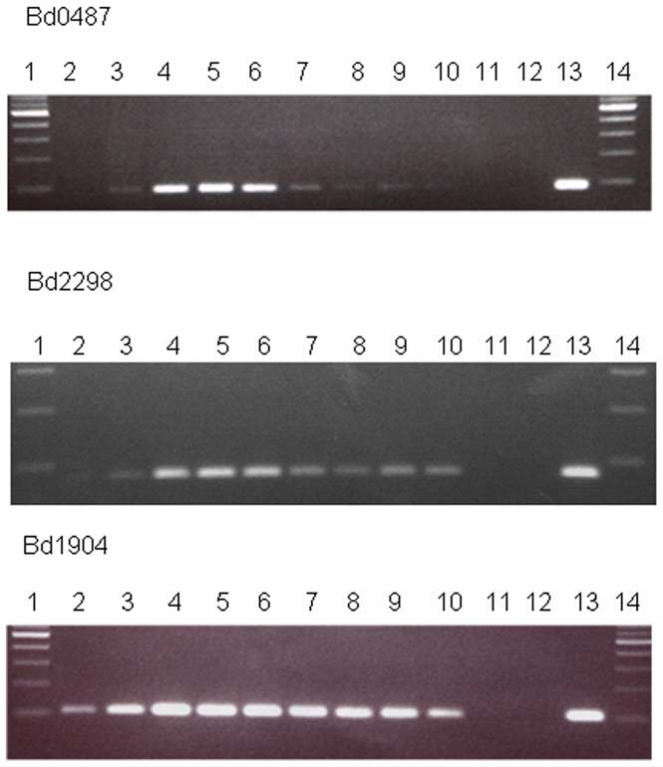
Transcription profiles of upregulated hypothetical genes across the predatory cycle. RT-PCR on total RNA prepared from identical volumes of *B. bacteriovorus* HD100 predator-prey *E. coli* S17-1 infection culture as the predatory infection proceeds across a time course. Primers were designed against three *Bdellovibrio*-specific but otherwise unknown function that specify proteins with signal sequences. These genes were shown by the array studies to be significantly up-regulated upon the shift from attack-phase to 30 minutes of predatory growth. Lanes 1 & 14 NEB 100bp ladder, 2: attack-phase 3: 15 minutes predation, 4: 30 minutes predation, 5: 45 minutes predation 6–9: 1,2,3,4 hours predation respectively 10: HI growth, 11: no template control 12: *E. coli* S17-1 only RNA control 13 *B. bacteriovorus* HD100 genomic DNA control.

Several *Bdellovibrio* genes of unknown function are significantly up-regulated on the shift from attack phase to predatory growth or HI growth. Some of these are unique to *Bdellovibrio bacteriovorus* genome, some are shared with the related *Bacteriovorax marinus* genome (available in draft assembled contigs at the Sanger Centre website http://www.sanger.ac.uk/Projects/B_marinus/) and some are conserved hypothetical genes in other bacterial genomes, particularly in other delta Proteobacteria. Some of the most highly up-regulated genes fell into this unknown function category. We took three of these genes (Bd0487, Bd1904 which are solely predatorily up-regulated and Bd2298 which is up-regulated in both HI and predatory growth) and studied their transcriptional pattern by RT-PCR across total RNA from a predatory timecourse of *E. coli* infection (Figure 6). All three were very highly significantly up-regulated upon 30 minutes predation with fold values of 7.4, 13.9 and 6.6 respectively. Two of the three (Bd1904 and Bd2298) have significant BLAST homology only with predatory *Bdellovibrio* and *Bacteriovorax* genomes and one, Bd0487 is found only in the *Bdellovibrio bacteriovorus* genome. All three genes are predicted to encode proteins with a confidently predicted signal peptide and thus products that could be periplasmically exported for predatory or other roles. As expected the RT-PCR timecourses indicate significant up-regulation in RNA from the attack-phase free living cells, to cells plus prey at 15 minutes and 30 minutes (Figure 6).

### Genes Up-Regulated under HI Conditions but Not at 30 Minutes Predation

This category contains a large number of genes because the HI cells are growing and replicating fully in artificial lab media but the predatory culture has only had 30 minutes of prey invasion so is just beginning the process of transcribing and translating the waves of predatory gene products that will later allow it to grow and replicate. Thus very many common pathways for *Bdellovibrio* primary metabolism, growth and division show up as specific only to HI dataset at this stage. As mentioned earlier, we chose the predatory timepoint of 30 minutes for our studies to ensure synchrony of infection and to allow us to characterise early predatory gene expression. It is however interesting to note that there are three gene clusters (Bd0828–Bd0838, Bd1473–Bd1483 and Bd2368–Bd2377) with *tonB*/*agl* homologies that are up-regulated in the HI, but not the predatory dataset, and that these operons do not include the Bd0412–420 gene cluster that was seen as up-regulated only in the predatory dataset. So although it is possible that, as for metabolic and growth division genes, the predatory *Bdellovibrio* may use these later in the predatory growth phase, they may represent discretely different TonB systems with different functions. As is seen in the next section below, examination of down regulation of attack-phase genes on the shift to predatory growth, lends some support to the idea of these defined uses of the different TonB gene products in HI versus predatory growth.

### Down-Regulated Genes on the Shift out of Attack Phase

Attack-phase is the non replicative highly motile phase of *Bdellovibrio* growth when cells are “seeking” encounters with prey cells to attach to and invade them. Genes that are in the dataset down-regulated from attack phase to HI or predatory growth, are those which are likely to be important in responding to the environmental pressures associated with the free-swimming phase and in initial prey location (HI cells do not need to actively locate prey to grow and reproduce). Consistent with this, a significant number of motility and chemotaxis genes are amongst the dataset down-regulated from attack phase to both HI and bdelloplast, reiterating the importance of motility and taxis in attack phase as previous work had indicated [2], [32]. The second largest group of down-regulated genes are those predicted to encode outer membrane proteins. The program pSORTB (version 2.0.4; http://www.psort.org/psortb/) predicts that 100 proteins, encoded by the *B. bacteriovorus* HD100 genome, localize to the outer membrane and greater than 60% of these are of unknown function. Many may have predatory-specific functions including mediating the physical attachment of *Bdellovibrio* to prey bacteria. Genes down-regulated in HI but not predatory conditions, versus attack-phase may be required in attack phase for the initial prey interaction and then further to stay in contact with the prey, but are not necessary for the HI growth phase. Little is known of the molecular nature of the initial physical contact between predator and prey and this dataset could give us some clues as to which outer membrane proteins could be involved. Further study of this could reveal the nature of prey specificity as well as reasons for the apparent inability of the system to generate simple *Bdellovibrio*-resistant prey mutants.

### Attack Phase Genes That Are Down Regulated on the Shift to Predatory but Not HI Growth

Our attack phase cells were recently liberated from prey but were suspended in prey-free medium, and “seeking prey” in our transcriptional studies. As discussed in the section above they would be expected to express prey-attachment genes, but some of these might only be required in the early minutes of prey outer membrane encounters, and possibly also genes encoding enzymes to breach the prey outer membrane which would be deleterious once inside the bdelloplast. Expression of such genes would already be being down-regulated once the *Bdellovibrio* had entered the prey periplasm.

Only 67 genes were significantly down-regulated only upon the shift to predatory growth in a prey periplasm and 163 genes down-regulated both in a shift to predatory growth or HI growth. After the hypothetical and flagellar genes next largest group of down-regulated genes in the shift to predatory growth only were Bd1476–1481, which are part of one of the 4 the *tonB*-like *agl* gene clusters mentioned above. This further suggests that TonB-like systems may be growth-phase specific. Only a small group of genes encoding membrane proteins (Bd3059, Bd3260 Bd2608, Bd2400 with a potential role in prey contact were in this predatorily down-regulated dataset. A small group of genes whose products may participate in breaching the outer layers of prey cells, and whose function may be finished by 30 minutes predation, are down-regulated. These include Bd0736 and Bd0737 encoding putative lipases, Bd0992 encoding a putative cell wall hydrolase, Bd3749 encoding a protease, Bd3857 encoding an alkaline serine protease. A gene encoding a homologue of the periplasmic adaptor protein CpxP that is involved in misfolded protein proteolysis in other Gram-negative bacteria [33] is also down-regulated. Two genes encoding transcriptional regulators Bd0931 and Bd1826 were down-regulated, along with a gene encoding a putative RNA binding protein Bd0339. These regulators may be involved in maintaining the non-replicative state of the attack phase cell or in expressing genes required for the highly aerobic environment in which it swims; a contrast to the less aerobic environment inside the prey periplasm. Considering what causes the attack-phase cell not to replicate, two other predatorily down-regulated genes are of interest: Bd1167 which encodes a coiled-coil crescentin like protein with actin-binding properties that may control cytoskeletal rearrangements, and rather surprisingly, chaperone protein gene *dnaK* (a gene which was not down-regulated in the shift to HI conditions). DnaK is well known in non-predatory bacteria for its chaperone activities, but interestingly its over production is also known to prevent cell-septation in *E. coli* and other bacteria and its relative level to that of DnaJ is important in septation [34], [35]. It is interesting that *Bdellovibrio* has 2 strong *dnaJ* homologues Bd1296 and Bd0677. Bd1296 expression is unchanged in the shift to predatory or HI growth but Bd0677 is 6-fold downregulated only from attack phase to HI. We hope to test in the future whether down-regulation of *dnaK* or *dnaJ* ratios in predatory cells relieves the septation block in *Bdellovibrio* attack-phase cells upon predation.

### Attack-Phase Genes That Are Down-Regulated in Both Predatory and HI Growth

The categories of down-regulated genes of *Bdellovibrio* as it leaves attack-phase fit with a view of the predatory bacterium ceasing flagellar motility as it settles into the periplasm of its prey and begins to receive prey-derived amino-acids or the longer slower HI cell (which expresses a few or no flagella on a several micron-long cell) forming a biofilm. The cyclic di-GMP signalling system is associated in bacteria with transitions from a motile to a sessile life style, the periplasmic and extracellular secretion of compounds and compartmentalised or bacterial-organelle-specific regulation- as for the bacterial flagellum [36]. *Bdellovibrio* has 5 predicted cyclic di-GMP producing GGDEF genes in its genome and 15 potential pilZ cyclic di-GMP binding receiver genes. Thus it is probably not surprising that one of the 5 GGDEF genes and three of the *pilZ* genes Bd2524, Bd2545 and Bd3100 are significantly down-regulated upon the shift from motile attack phase to sessile predation or HI growth.

The switch from attack phase to either HI growth or the predatory invasion of prey brings to the *Bdellovibrio* a change from a “hungry” non-replicative state to growth at the expense of prey-derived molecules, or media and ultimately replication and septation. Transcription of *dksA* Bd3519 drops significantly on the exit from attack-phase; DksA is important in the stringent response to amino-acid starvation in bacteria such as *E. coli* [37]. It associates with ribosomes and when uncharged tRNAs are sensed at those ribosomes, ppGpp is synthesised to down regulate rRNA synthesis in the stringent response. As the *Bdellovibrio* are moving from a state of relative starvation in attack phase to a time of amino-acid plenty, it makes sense for the ppGpp-mediated suppression of rRNA synthesis to be lifted to match the large increase in ribosomal protein and ribosomal RNA synthesis that is seen in the highly up-regulated gene-sets for predatory and HI growth. After this ribosomal synthesis leading to more protein synthesis, *Bdellovibrio* septation will ultimately occur. One hint of the regulation of this process comes in the down-regulation of Bd0464 which encodes a DivIVA cell division homologue. DivIVA is known in Gram-positive bacteria as regulator of septum formation in *Bacillus* in which localises to the poles of the cell, and which organises filamentous budding in *Streptomyces* [38]. It is possible that the DivIVA homologue has a direct role in negatively regulating septum formation and cell division in the attack-phase cell and thus its depletion at the 30 minute stage allows *Bdellovibrio* to prepare to septate as it has now entered its replicative phase inside prey or in HI growth. We are currently studying this microscopically.

Past research by Thomashow [39] and further studies by Jurkevitch [40] highlighted a locus, the *hit* locus, genes Bd0108–Bd0121 where mutations were often, but not compulsorily found in *Bdellovibrio* strains that were growing as HI. Mutations were described for some HI strains, in gene Bd0108 that abolished or truncated the reading frame, and Rendulic and co-workers [1] commented that the other genes in the cluster could encode part of a flp pilus system. Interestingly the Bd0108–0121 cluster is significantly down-regulated in the shift from attack phase to predatory growth. (Its expression does drop in the shift to predatory from attack-phase also but not sufficiently to be called as significant in our analyses.

### Conclusions

**Figure 7:**
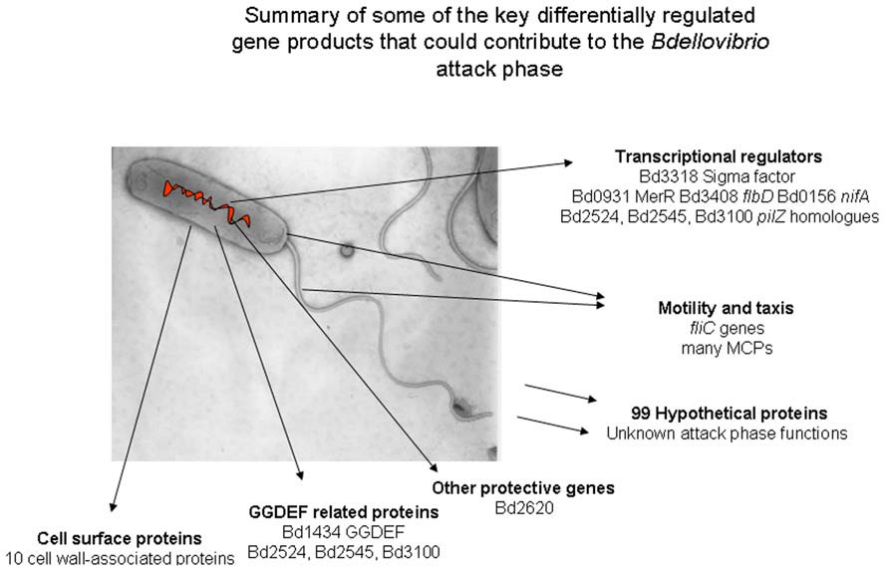
Gene expression key to attack phase. Visual summaries of key gene expression characteristics of the attack phase of *Bdellovibrio* cells, as implied by genes highly expressed In the attack phase dataset, but downregulated in the other two datasets.

**Figure 8:**
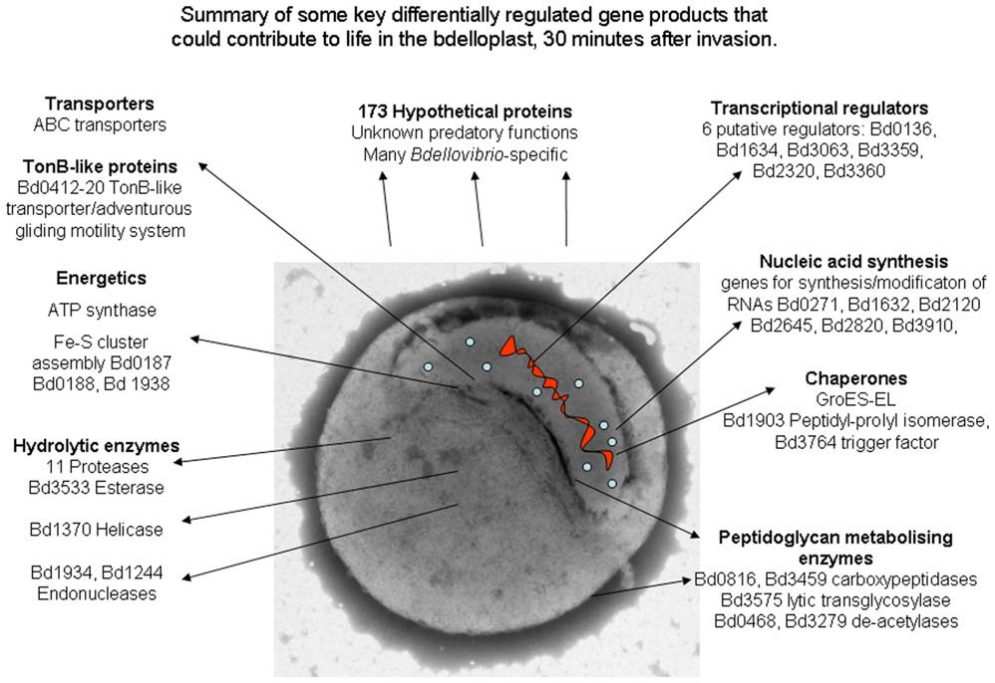
Gene expression key to 30 minutes predation. Visual summaries of key gene expression characteristics at 30 minutes of predatory growth by *Bdellovibrio* cells, as implied by genes highly expressed in the predatory dataset, but downregulated in the other two datasets.

**Figure 9:**
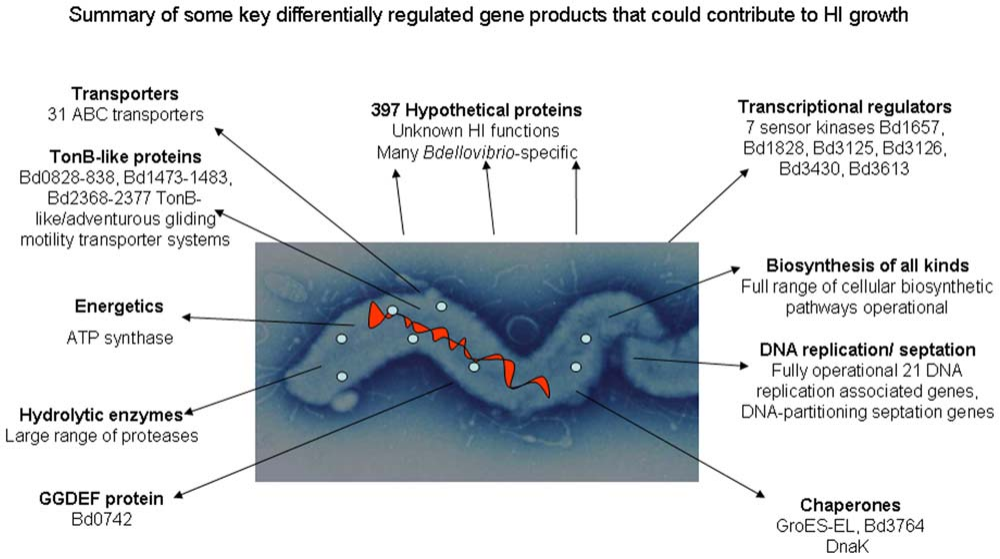
Gene expression key to HI growth phase. Visual summaries of key gene expression characteristics of the HI growth phase of *Bdellovibrio* cells, as implied by genes highly expressed in the HI dataset, but downregulated in the other two datasets.

The comparison between *Bdellovibrio* gene expression in the “hungry, prey-hunting” attack-phase, the early prey invasion and establishment phase and the prey-independent axenic growth phase (which may mimic biofilm growth of *Bdellovibrio* in aquatic environments) give, for the first time a genome-wide view of the expression of *Bdellovibrio* genes that are required for these diverse lifestyles, and account for the seemingly large genome size of this predatory bacterium [1]. Our data also indicate that “attack-phase” genes whose products are required for productive interactions with prey bacteria are rapidly down-regulated upon predatory invasion and that the HI- axenic growth phase does have its own gene expression signature, not simply encompassing genes required for growth and cell replication. We have attempted to summarise key differentially expressed genes in 3 diagrams for attack phase (Figure 7), 30 minutes predatory growth (Figure 8) and HI growth phase (Figure 9).

Although transcriptional profiling has been commonplace for many bacteria previously, it has not been applied to the *Bdellovibrio* : *E. coli* predator prey system before, so previous experiments to study predator-prey interaction, or axenic HI growth genes have relied upon random transposon mutagenesis [41], [42] or targeted “hunches” (including our own!) for gene inactivation. One previous study by Jurkevitch and co-workers [10] studied 196 proteins comparing 2D gel protein profiles for attack-phase *Bdellovibrio* to that of *Bdellovibrio* growing host-independently over a timecourse. Our transcriptional data support the majority of their findings and many of the earlier “hunches” and some of the mutagenesis and selection studies of the Kadouri and Tudor labs (which we discuss below). However they also allow more extensive hypotheses, about the roles of different systems and pathways, and of *Bdellovibrio*-specific genes in predation, or HI growth, to be developed and tested experimentally by the *Bdellovibrio* community.

Firstly looking at gene expression patterns that are supportive of earlier *Bdellovibrio* research- our data support those from the 2D gel study of Jurkevitch and co-workers that show up-regulation of chaperonins, translation elongation factors and the general secretory pathway genes and other specific genes including hypothetical protein genes such as Bd1100, and nucleotide metabolism gene Bd1553, in HI compared to attack-phase growth. It was also clear that flagellar and chemotaxis genes were down-regulated, comparing *Bdellovibrio* growth in attack-phase to that in either predatory or HI mode, confirming our study that flagellar motility was important for prey encounters [2] and the work of the Kadouri lab [42]. It was clear that genes encoding peptidoglycan modification and degradation were up-regulated in predatory cells compared to attack-phase, including a peptidoglycan deacetylase gene Bd3279 and D-ala-D-ala carboxypeptidase activities. These enzyme activities were monitored in bdelloplasts in two papers from 1978 by Thomashow and Rittenberg [27], [28] and it is great to see genes with a cognate expression pattern emerging 30 years after their elegant biochemical research was published. We are currently following up the predatory activities of these genes by mutagenesis.

Another clear finding, from our HI-specific up-regulated gene expression dataset, was that three of the four potential *tonB*-like/adventuorous gliding motility operons in the *Bdellovibrio* genome (Bd0828–Bd0838, Bd1473–Bd1483 and Bd2368–Bd2377) were significantly up-regulated only during HI growth, with the Bd1473–1483 operon being down-regulated in the transition from attack-phase to 30 minutes predation. Two genes in this latter operon have been implicated, in a mariner mutagenesis screen and study of HI *Bdellovibrio*, by the Kadouri lab in predation of biofilms [42] and our data here supports the idea that *Bdellovibrio* may be using gliding motility or another TonB-dependent transport process for growth in HI biofilms.

Tudor and co-workers [41] used mini-Tn5 mutagenesis to isolate 14 predatory genes harbouring the transposon in their coding sequence, and although our transcriptional study only covers genes that are up-regulated early at 30 minutes of predation (not in the whole 3 hours when predatory mutants may be affected) we find that three of the Tudor's predatory genes with a hypothetical annotation Bd2033, Bd3170 and Bd3518 and two of the protease genes Bd2428 and Bd3534 are up-regulated in predatory growth compared to attack-phase. Pineiro and co-workers [43] mutagenised a *Bdellovibrio* nudix hydrolase gene Bd0714 but found that it had no effect upon the predatory growth of the bacteria, our studies support this showing that in comparison to attack-phase growth Bd0714 was up-regulated in HI growth, but showed no change in expression at 30 minutes post-predation.

Our data do support the idea of a “belt and braces” situation, where multiple copies of gene families have evolved by gene duplication, to encode important processes in the predatory or HI lifestyles. For example the three operons predicted to encode related TonB-like systems that are up-regulated in the HI lifestyle and seven cell-wall anchor family protein genes (belonging to an expanded *Bdellovibrio* family of genes, that are not present in closely-related non-predatory delta-proteobacteria such as *Geobacter*), that were down-regulated specifically in the HI lifestyle.

Such differential gene regulation is good evidence of the HI phenotype being a distinct phase in the lifecycle. That genes encoding cell wall proteins, specific to attack phase, (many probably being involved in the attachment to, or entry into prey) were down-regulated specifically in the HI phase and that many other genes are differentially regulated in just HI or predatory data sets (and not in both) suggests that HI growth does not just “accidentally mimic” the intracellular predatory phase, indicating that the presence of the prey is clearly being detected in the latter *Bdellovibrio* but not the former.

It is possible that the HI phenotype is turned on in situations such as biofilms so different environmental stresses are experienced by the close bacterial living there, requiring HI-specific gene induction, and there is recent evidence to support this [44], [45], [46]. If *Bdellovibrio* were to grow as HI in biofilms, it could partially explain the lack of complete amino acid pathways seen in the genome [1] as they may be taking vital amino acids up from surrounding cells which have lysed. Our finding that the *hit* locus genes Bd0108–Bd0121 are down-regulated upon the shift to HI growth re-emphasises the need for renewed investigations into their role, some of which we are undertaking.

Our up-regulated gene-sets have highlighted the employment of some “routine” housekeeping systems such as the Tat and Sec transporters in predatory and HI growth processes. Also some more tantalising suggestions have come forward including the testable possibilities that *dnaK* levels may contribute to the replication-septation arrest seen in attack-phase *Bdellovibrio*.

In our predatorily up-regulated, predatosome gene set, we see genes whose products (such as peptidoglycan modifiers) may facilitate the remodelling of the prey cell periplasm into a home for the *Bdellovibrio*. In addition, more than 170 genes encoding hypothetical proteins are in the predatosome, many of which are unique to the bdellovibrios-and-like-organisms, and now high throughput mutational analyses can be applied to test their roles in the bdelloplasting process. Targeting these predatosome genes, rather than the whole genome, is important in a bacterium like *Bdellovibrio* where plasmid replication is difficult and complementation and inhibitory RNA studies are challenging and where all mutations must currently be made by conjugation.

Eventually a systems biology approach to mapping the function of all “predatosome” genes and their interaction with the core housekeeping genes of *Bdellovibrio* will be achieved, but for the present we hope that the data from our studies will encourage the field into further detailed understanding of the predatory life of this fascinating bacterium, the nature of the gene complement it anciently acquired to become predatory, and that which it retained for axenic growth and when that growth phase is naturally induced. Furthermore an understanding of the HI growth mode, and how to control it, may allow us to design therapeutic, obligately-predatory *Bdellovibrio* for medical applications against Gram-negative pathogens.

## Supporting Information

Figure S1 Expression data by category. Excel file showing fold- differential expression for each gene set. Fold-Change 1 was calculated by calculating the fold-change of each paired datapoint and averaging all of these for every gene. Fold-Change 2 was calculated by averaging the normalised expression of each datapoint for a gene and calculating the fold-change between experimental conditions for each gene. The T-test shown was a paired, 2 tailed test for each comparative experimental condition.
(1.16 MB XLS)
